# Effectiveness of Antiseizure Medication Triple Therapy in Patients With Glioma With Refractory Epilepsy: An Observational Cohort Study

**DOI:** 10.1212/WNL.0000000000206852

**Published:** 2023-04-04

**Authors:** Pim B. van der Meer, Linda Dirven, Marta Fiocco, Maaike J. Vos, Mathilde C.M. Kouwenhoven, Martin J. van den Bent, Martin J.B. Taphoorn, Johan A.F. Koekkoek

**Affiliations:** From the Leiden University Medical Center (P.B.v.d.M., L.D., M.F., M.J.V., M.J.B.T., J.A.F.K.); Haaglanden Medical Center (P.B.v.d.M., L.D., M.J.V., J.A.F.K.), The Hague; Amsterdam University Medical Centers (M.C.M.K.); and Erasmus Medical Center (M.J.v.d.B.), Rotterdam, the Netherlands.

## Abstract

**Background and Objectives:**

Approximately 10% of patients with glioma with epilepsy need antiseizure medication (ASM) triple therapy due to refractory epilepsy. The aim of this study was to evaluate whether levetiracetam combined with valproic acid and clobazam (LEV + VPA + CLB), a frequently prescribed triple therapy, has favorable effectiveness compared with other triple therapy combinations in patients with glioma.

**Methods:**

This was a multicenter retrospective observational cohort study. The primary outcome was the cumulative incidence of time to treatment failure for any reason, from the start of ASM triple therapy treatment. The secondary outcomes included cumulative incidences of the following: (1) time to treatment failure due to uncontrolled seizures; (2) time to treatment failure due to adverse effects; and (3) time to recurrent seizures. Patients were followed up for a maximum duration of 36 months.

**Results:**

Of 1,435 patients in the original cohort, 90 patients received ASM triple therapy after second-line ASM treatment failure due to uncontrolled seizures. LEV + VPA + CLB was prescribed to 48% (43/90) and other ASM triple therapy to 52% (47/90) of patients. The cumulative incidence of treatment failure for any reason of LEV + VPA + CLB did not statistically significantly differ from that of other ASM triple therapy combinations (12 months: 47% [95% CI 31%–62%] vs 42% [95% CI 27%–56%], *p* = 0.892). No statistically significant differences for treatment failure due to uncontrolled seizures (12 months: 12% [95% CI 4%–25%] vs 18% [95% CI 8%–30%], *p* = 0.445), adverse effects (12 months: 22% [95% CI 11%–36%] vs 15% [95% CI 7%–27%], *p* = 0.446), or recurrent seizures (1 month: 65% [95% CI 48%–78%] vs 63% [95% CI 47%–75%], *p* = 0.911) were found.

**Discussion:**

LEV + VPA + CLB might show equivalent effectiveness compared with other ASM triple therapy combinations in patients with glioma.

**Classification of Evidence:**

This study provides Class III evidence that for patients with glioma with refractory epilepsy on triple therapy ASMs, LEV + VPA + CLB demonstrated similar effectiveness and tolerability compared with other ASM triple therapy combinations.

Epileptic seizure management is an important aspect in the disease trajectory because preoperative seizures occur in up to approximately 75% of patients with diffuse gliomas.^1^ Antiseizure medication (ASM) treatment is indicated once a first seizure has occurred.^2^ However, drug-resistant epilepsy (defined by the International League Against Epilepsy as patients without adequate seizure control after ≥2 trials with ASMs either in monotherapy or in combination) occurs in approximately 15% and up to 40% of patients with glioblastoma and grade 2 glioma, respectively.^3,4^ Benzodiazepines and particularly clobazam (CLB) are commonly prescribed add-on ASMs in drug-resistant epilepsy likely due to their ease of administration and ease of use. CLB does not require a careful titration and only needs to be taken once or twice a day.^5^ CLB is a 1.5-benzodiazepine (nitrogen atoms are located at positions 1 and 5 of the diazepine ring) and is believed to have various mechanisms of action, but the major effect is the potentiation of γ-aminobutyric acid (GABA)ergic neurotransmission. It has better tolerability compared with traditional 1.4-benzodiazepines. Due to its unique 1.5-pharmacological profile, it is believed to give it a broader spectrum of anticonvulsive activity and possible synergistic efficacy when used with other ASMs.^6^ CLB is frequently prescribed in patients with brain tumor, but only 1 study evaluated its efficacy (30% seizure freedom within 6 months of initiation of CLB) and tolerability (6% experiencing intolerable adverse effects) as add-on ASM in the brain tumor population.^7,8^ Methodological issues, however, such as not taking into account the competing risk of death and lack of a comparison group, hamper reliable interpretation of results. In patients with non–brain tumor–related epilepsy (BTRE), CLB seems to perform reasonably well (12-month retention of approximately 60%–80% in patients with refractory epilepsy) compared with other ASMs, but large comparative efficacy trials are lacking.^9,10^ Four double-blind placebo-controlled randomized controlled trials have been conducted in the past decades, representing only 197 patients, evaluating CLB as add-on in patients with non-BTRE drug-resistant epilepsy. CLB may be effective in reducing seizure frequency in focal-onset seizures, but it should be noted that this finding is based on very low-quality evidence, and all 4 included studies have an unclear risk of bias due to insufficient reporting of methodological details.^11^

Recently, we demonstrated in a large multicenter retrospective observational study that first-line monotherapy levetiracetam (LEV) has favorable efficacy compared with valproic acid (VPA), 2 commonly prescribed ASMs in the glioma population.^12^ This finding is supported by a recent systematic review in which monotherapy LEV had the most favorable efficacy along with pregabalin and phenytoin. However, the latter 2 ASMs were less well tolerated, reflected in higher treatment failure due to adverse effects rates.^8^ If seizures are not adequately controlled on ASM monotherapy, the combination of LEV with VPA has favorable efficacy compared with other ASM dual therapy combinations with either LEV or VPA.^13^ In approximately 10% of patients with glioma with epilepsy treated with ASMs, a third ASM is prescribed with the aim of reaching adequate seizure control.^12^ With approximately 30 different ASMs available for use in clinical practice, more than 4,000 triple-therapy combinations can be made, complicating the evaluation of ASM triple therapy treatment.^14^ Despite this plethora of combinations, a frequently prescribed triple therapy combination is LEV combined with VPA and CLB (LEV + VPA + CLB) because CLB is added to the dual therapy combination of LEV with VPA. The aim of this study was to evaluate whether LEV + VPA + CLB has favorable effectiveness in patients with glioma with refractory epilepsy compared with other ASM triple therapy combinations.

## Methods

### Study Population and Procedures

A more extensive description of the methodology has been previously published.^12^ This was a multicenter retrospective observational study, and all consecutive patients with a histologic diagnosed World Health Organization (WHO) grade 2–4 diffuse glioma according to the WHO 2016 classification of CNS tumors,^15^ between January 1, 2004, and January 1, 2018, and had undergone biopsy or surgical (re)resection were included. Participating centers were Erasmus Medical Center, Haaglanden Medical Center, and Amsterdam University Medical Centers (location VUMC). Patients receiving first-line monotherapy treatment with either LEV or VPA were included in the original cohort (n = 1,435).^12^ Patients showing treatment failure due to uncontrolled seizures on their first-line LEV or VPA and receiving ASM dual therapy treatment were included in the subsequent study (n = 355).^12,13^ Patients showing treatment failure on their ASM dual therapy treatment due to uncontrolled seizures and receiving ASM triple therapy treatment subsequently were included in this study. We compared 2 groups: LEV + VPA + CLB vs other triple therapy combinations. Patients were excluded if the add-on ASM was prescribed with the intention for a limited period of time (a maximum term of 3 months). The following baseline (i.e., from the starting date of ASM triple therapy initiation) information was collected from the patients' charts: sociodemographic data, tumor-specific information, data on antitumor treatment, radiologic progressive disease (during treatment failure due to uncontrolled seizures) based on the Response Assessment in Neuro-Oncology criteria,^16^ and ASM treatment information. To assess potential dose escalation and/or dose reduction differences between the 2 groups during ASM treatment failure, the ASM load was calculated for each patient because not all ASMs have similar defined daily dosages (DDDs). ASM load is defined as the sum of the ratio between the prescribed daily dosage and the DDD of each individual ASM included in the ASM treatment combination (eTable 1, links.lww.com/WNL/C610).^17^ For instance, the DDD of CLB is 20 mg and of LEV and VPA 1,500 mg. In case a patient is prescribed 10 mg CLB, 2,500 mg LEV, and 2,000 mg VPA each day, the ASM load is 3.5 ([10/20] + [2,500/1,500] + [2,000/1,500]).

### Outcomes

Time to treatment failure for any reason, a measure for ASM effectiveness that includes efficacy and tolerability,^18^ was the primary outcome. It was estimated from the time of initiation of ASM triple therapy until treatment failure, death, lost to follow-up, or reaching the end of study date (patients were followed up for a maximum duration of 36 months). We defined ASM treatment failure as the addition, withdrawal, or replacement of an ASM. We considered the following events not as treatment failure: the addition of an ASM pro re nata (i.e., when required), the use of approved ASMs outside epilepsy (e.g., carbamazepine as treatment for trigeminal neuralgia), changing the dosage of the initial ASM triple therapy combination, the addition of a temporary primary prophylactic ASM during a perioperative period, the replacement of ASMs in the end-of-life phase with a nonoral route of administration (e.g., buccal clonazepam) due to dysphagia, or poor adherence <1 week. Evaluated secondary outcomes were as follows: (1) time to treatment failure for specific reasons of treatment failure (i.e., adverse effects, uncontrolled seizures, withdrawal due to remission of seizures, or other reasons); (2) time to recurrent seizures, a measure for efficacy, similarly estimated as time to treatment failure (a maximum duration of follow-up of 36 months), until recurrent seizures, death, treatment failure (with the exception of treatment failure due to uncontrolled seizures), lost to follow-up, or reaching the end of study date; and (3) tolerability, which we defined according to the severity (grades 1–5) of adverse effects leading to ASM discontinuation (i.e., intolerability was based on clinical judgment of the treating physician) based on the Common Terminology Criteria for Adverse Events, version 5.0.^19^ Of each intolerable adverse effect, it was evaluated whether it improved after ASM discontinuation based on laboratory results and information reported by clinicians in the patients' charts, in a period of approximately 1–2 months. Improvement of the intolerable adverse effect(s) after discontinuation of the ASM was seen as a valid reason to regard the suspected discontinued ASM as a likely causative (contributing) factor of the intolerable adverse effect(s).^20^

### Statistics

Time to treatment failure and time to recurrent seizure were analyzed with a competing risk model comparing the cumulative incidences of LEV + VPA + CLB with other ASM triple therapy.^21^ The following 3 competing risk models were applied: (1) time to treatment failure for any reason (2 competing events: outcome of interest and death); (2) time to treatment failure for specific reasons of treatment failure (5 competing events: treatment failure due to adverse effects, uncontrolled seizures, withdrawal due to remission of seizures, other reasons, and death); (3) time to recurrent seizure (3 competing events: outcome of interest, treatment failure before a recurrent seizure has occurred, and death). We reported the cumulative incidences at 1, 12, and/or 36 months after ASM initiation in the main text (including 95% CI) because we regarded these time points as clinically most relevant. The Gray test was applied to assess the difference between the cumulative incidences.^22^ Baseline demographic characteristics were analyzed with the χ^2^ test, and ASM load during treatment failure was analyzed with the independent *t* test. A power calculation and sample size estimation was performed for the original cohort only,^12^ but not for this study. Therefore, statistical analyses based on our small cohort should be regarded mainly as descriptive. All statistical tests were performed with SPSS software, version 25.0, and R software.^23,24^ Statistical tests for the competing risk models were performed with R by using the cmprsk library.^21^ Statistical significance was set at a *p* value of <0.05. *p* Values were only reported for time to treatment failure, time to recurrent seizure, baseline demographic characteristics, and ASM load during treatment failure and not for other comparisons because of the descriptive nature of our study and to avoid (statistical) inference based on reported *p* values.

### Standard Protocol Approvals, Registrations, and Patient Consents

The study protocol was approved by the medical ethics committee of each institution, and informed consent of included patients was obtained according to the institutions policy.

### Data Availability

Data supporting the findings of this study are available from the corresponding author on reasonable request.

## Results

### Patient Characteristics

The baseline characteristics of the included patients are summarized in Table 1. A total of 90 patients received ASM triple therapy and were included in this study. LEV + VPA + CLB was prescribed to 48% (43/90) and other ASM triple therapy combinations to 52% (47/90) patients equaling 22 different combinations (eTable 2, links.lww.com/WNL/C610). Patients in the LEV + VPA + CLB group had significantly more often a Karnofsky performance status ≥70 and a history of a psychiatric disease.

**Table 1:**
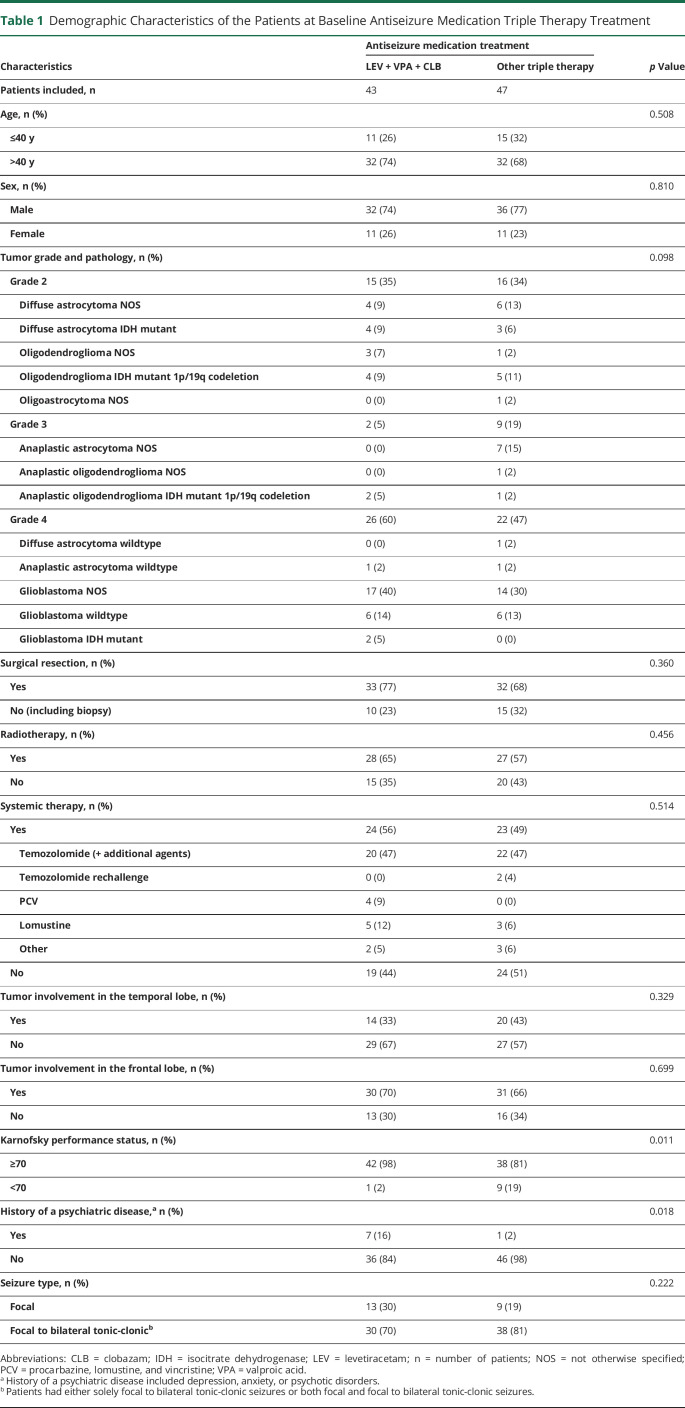
Demographic Characteristics of the Patients at Baseline Antiseizure Medication Triple Therapy Treatment

### Time to Treatment Failure

**Figure 1:**
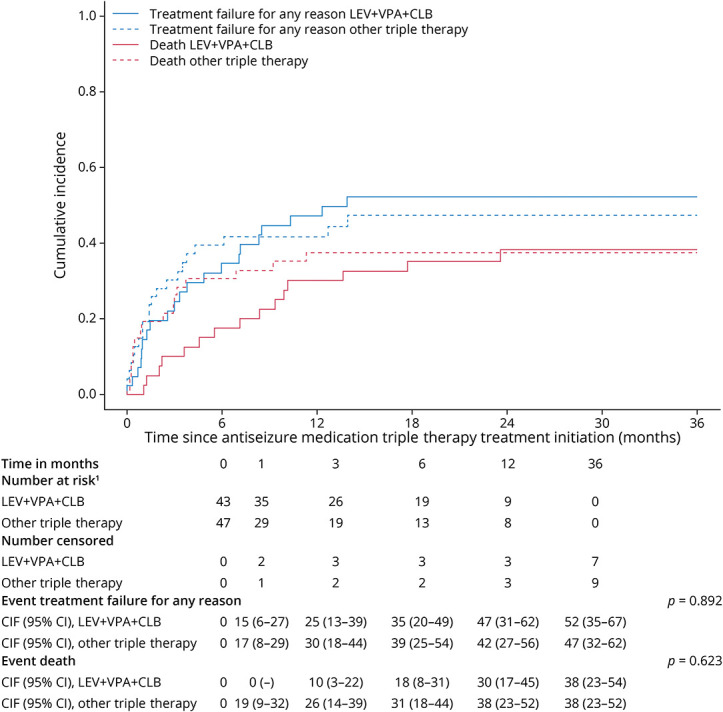
Time to Treatment Failure for Any Reason: LEV + VPA + CLB vs Other Triple Therapy ^1^Number of patients at risk refers to the number of patients who have not experienced an event (i.e., the event treatment failure of the event death) at that particular time point (e.g., 3 months) and who are still at risk of experiencing an event (i.e., not censored). CIF = cumulative incidence function; CLB = clobazam; LEV = levetiracetam; n = number of patients; VPA = valproic acid.

During the 36 months follow-up, a total of 49% (21/43) patients who used LEV + VPA + CLB and 45% (21/47) who used other triple therapy combinations showed treatment failure. The cumulative incidence of treatment failure for any reason did not significantly differ between LEV + VPA + CLB and other triple therapy combinations (12 months: 47% [95% CI 31%–62%] vs 42% [95% CI 27%–56%], *p* = 0.892; Figure 1). Neither were there differences for treatment failure due to uncontrolled seizures (12 months: 12% [95% CI 4%–25%] vs 18% [95% CI 8%–30%], *p* = 0.445), adverse effects (12 months: 22% [95% CI 11%–36%] vs 15% [95% CI 7%–27%], *p* = 0.446), other reasons (12 months: 10% [95% CI 3%–22%] vs 7% [95% CI 2%–17%], *p* = 0.924), or withdrawal due to remission of seizures (36 months: 5% [95% CI 1%–16%] vs 2% [95% CI 0%–11%], *p* = 0.564; eTable 3, links.lww.com/WNL/C610).

The mean ASM load of LEV + VPA + CLB was significantly higher compared with other triple therapy combinations during treatment failure due to uncontrolled seizures (4.33 [SD 1.03] vs 3.21 [SD 0.48] ASM load, *p* = 0.029), but no significant differences were found between LEV + VPA + CLB and other triple therapy combinations for treatment failure due to adverse effects (2.89 [SD 0.54] vs 3.42 [SD 0.58] ASM load, *p* = 0.062). Radiologic progressive disease (i.e., tumor progression) during treatment failure due to uncontrolled seizures was present in 40% (2/5) of patients who used LEV + VPA + CLB compared with 13% (1/8) who used other triple therapy combinations.

### Time to Recurrent Seizure

**Figure 2:**
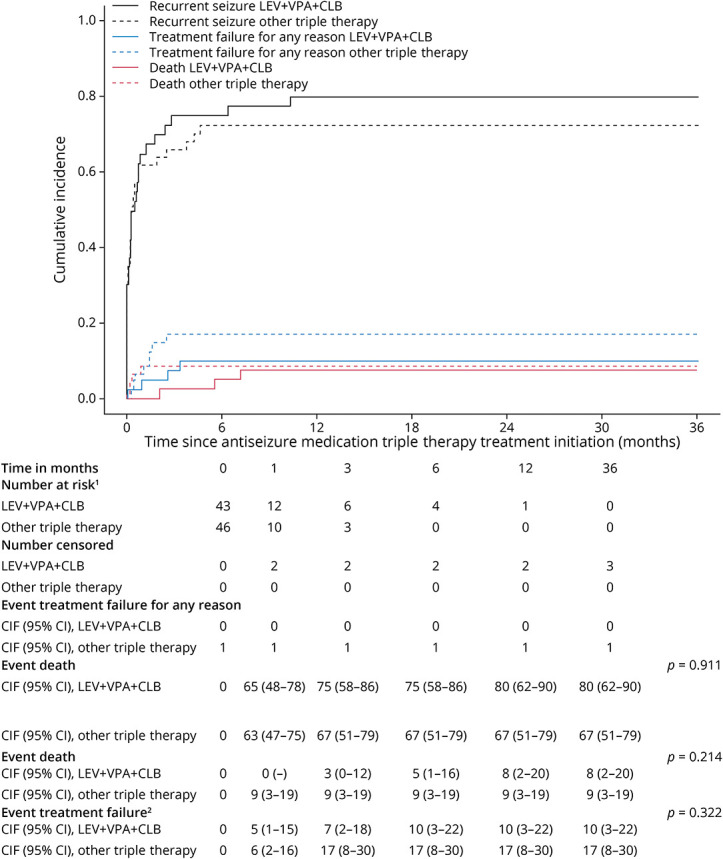
Time to Recurrent Seizure: LEV + VPA + CLB vs Other Triple Therapy ^1^Number of patients at risk refers to the number of patients who have not experienced an event (i.e., the event treatment failure of the event death) at that particular time point (e.g., 3 months) and who are still at risk of experiencing an event (i.e., not censored). ^2^Patients who experienced treatment failure (due to adverse effects, withdrawal due to remission, or other reasons) before experiencing their recurrent seizure can no longer experience a recurrent seizure on their first-line monotherapy LEV or VPA, and therefore, treatment failure was handled as competing risk. CIF = cumulative incidence function; CLB = clobazam; LEV = levetiracetam; n = number of patients; VPA = valproic acid.

Already 1 month after initiation of ASM triple therapy, most of the patients had experienced a recurrent seizure, but no significant differences for the cumulative incidences of a recurrent seizure were found between LEV + VPA + CLB and other triple therapy combinations (1 month: 65% [95% CI 48%–78%] vs 63% [95% CI 47%–75%], *p* = 0.911; Figure 2).

### Intolerable Adverse Effects

Sixteen intolerable adverse effects occurred in 10/43 patients who used LEV + VPA + CLB, while there were 9 intolerable adverse effects in 8/47 patients who received other triple therapy combinations (Table 2). CLB was discontinued in 5 (50%, in all 5 due to somnolence; eTable 4, links.lww.com/WNL/C610), VPA in 3 (30%), and LEV in 2 (20%) of the 10 patients who used LEV + VPA + CLB. The most common intolerable adverse effects in LEV + VPA + CLB and other triple therapy combinations were somnolence (6/16, 38%) and decreased platelet count (2/9, 22%), respectively. One patient in both the LEV + VPA + CLB and other triple therapy combination groups experienced a grade 3 or 4 adverse effect (6% vs 11%). In patients using LEV + VPA + CLB, 75% (12/16) of adverse effects improved vs 44% (4/9) in patients using other triple therapy combinations.

**Table 2:**
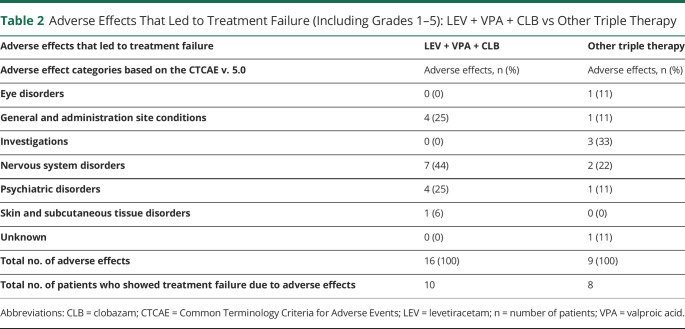
Adverse Effects That Led to Treatment Failure (Including Grades 1–5): LEV + VPA + CLB vs Other Triple Therapy

### Classification of Evidence

This study provides Class III evidence that for patients with glioma with refractory epilepsy on triple therapy ASMs, LEV + VPA + CLB demonstrated similar effectiveness and tolerability compared with other ASM triple therapy combinations.

## Discussion

This study aimed to compare the effectiveness of triple ASM treatment in patients with glioma with drug-resistant epilepsy, particularly LEV + VPA + CLB vs other ASM triple therapy combinations. No differences were found between the 2 studied triple therapy groups on efficacy or tolerability outcomes. On the contrary, the ASM load at the moment of treatment failure due to uncontrolled seizures was significantly higher for LEV + VPA + CLB, meaning dose escalation was probably less optimal in the other ASM triple therapy combinations, possibly increasing (prematurely) treatment failure due to uncontrolled seizures in the latter group. This might be partly explained by the difference in ease of administration because many ASMs need a more careful titration, and dose escalation is more slow compared with that in CLB. Altogether, the combination of LEV + VPA + CLB might perform equivalent compared with other ASM triple therapy combinations in patients with glioma. The addition of a third ASM to the treatment regimen might help to a limited extent in this difficult-to-treat population.

The cumulative incidence of patients who used LEV + VPA + CLB for a recurrent seizure was 75% (i.e., 25% seizure freedom) and treatment failure due to adverse effects was 17% at 6 months follow-up, of which approximately half was believed to be attributable to CLB by the treating physician, given CLB was discontinued. Efficacy and tolerability in our study were comparable with those in the study conducted by Brahmbhatt et al.,^7^ who found a seizure freedom of 30% at 6 months of follow-up and treatment failure due to adverse effects of 6% in patients with glioma with epilepsy who received add-on CLB and of whom most received ASM triple therapy. When comparing seizure freedom (12%–20%) and treatment failure due to adverse effects (8%–19%) after 3 months of follow-up in patients with non-BTRE receiving CLB as add-on,^11^ CLB does seem to perform quite similar in patients with glioma. Most of the patients with glioma in our cohort experienced a recurrent seizure within 1 month, while according to our definition, this implied treatment failure in only a minority of these patients. Given this cohort entails a population with drug-resistant epilepsy, having recurrent seizures seems to be more accepted by both the treating physician and the patient and does not necessarily lead to a change in ASM treatment regimen.

Choice of a particular ASM treatment regimen should not only depend on efficacy, but drug-related properties including pharmacokinetics, tolerability, safety, drug interactions, and ease of administration are of importance as well. CLB is generally considered as a safe ASM in (non-)BTRE, with dose-dependent adverse effects and severe adverse effects being very rare, and with an incidence rate of 1.6 per 1,000 person-years, the risk of benzodiazepine dependence low.^5,25^ No enzyme-inducing or enzyme-inhibiting properties have been found for CLB, and the drug levels of other ASMs did not change when CLB was added in pharmacokinetic studies. Common adverse effects include somnolence, dizziness, and ataxia.^5^ The additional anxiolytic properties of CLB might be a favorable side effect because approximately 25% of patients with glioma experiences symptoms of anxiety.^26^

It is the first time that a fixed and regularly prescribed combination of 3 ASMs is examined in patients with BTRE. The major mechanism of action of CLB is potentiation of GABAergic neurotransmission, which is a mechanism of action for VPA as well. Because rational polytherapy advises to combine ASMs with different mechanisms of action, CLB might not be the best choice to combine with LEV + VPA. For example, perampanel (PER) and lacosamide (LCM) might serve as efficacious add-on ASMs,^8^ being a noncompetitive α-amino‐3‐hydroxy‐5‐methyl‐4‐isoxazolepropionic acid glutamate receptor antagonist and^27^ voltage-gated natrium channels inactivator, respectively.^28^ PER combined with LEV does not seem to affect clearance and neither increased psychiatric adverse effects, such as agitation (the most intolerable adverse effect in LEV glioma patients),^12^ compared with other ASM combinations.^29^ Altered glutamate homeostasis seems to play an important role in the epileptogenesis in gliomas, making PER a rational treatment choice.^30^ LCM showed a synergistic effect with LEV and a tendency toward synergism with VPA in preclinical models.^31^ Because LCM has the advantage of having no interactions with other (antineoplastic) drugs and of a quick titration, unlike, for example, lamotrigine, it is considered a suitable (add-on) ASM in the glioma population. Our cohort is based on triple therapies prescribed in the past 2 decades. We suspect prescribed triple therapies in the last few years differ (partly) from previously prescribed triple therapies and think the proportion of LCM as add-on ASM in the triple therapy regimens is larger, while CLB might be smaller.

Given only 10% of patients with glioma with epilepsy need ASM triple therapy,^12^ recruiting a sufficiently large sample of patients that is needed to provide reliable results on the effectiveness of a specific ASM combination remains a major challenge. Due to the small cohort, we were not able to use matching techniques or multivariable regression analysis to take confounders into account. Therefore, (unknown) confounders were not equally distributed across the 2 studied treatment groups and could have influenced the outcomes. In a retrospective study design combined with a patient population with refractory epilepsy, time to recurrent seizure seems the best efficacy outcome despite its limitations because at 3 months, the number of patients at risk was already low. Ideally, when evaluating the comparative efficacy of ASMs in patients with refractory epilepsy (meaning a high-seizure frequency), prospectively assessed outcomes assessing seizure severity should be included.

In this retrospective observational study, LEV + VPA + CLB treatment in patients with epileptic refractory glioma might show similar effectiveness compared with other ASM triple therapy combinations. Prospective studies are needed to determine which ASMs are the most effective and tolerable add-on treatment options for patients with glioma with refractory epilepsy.

## Glossary

ASM: antiseizure medication
BTRE: brain tumor–related epilepsy
CLB: clobazam
DDD: defined daily dosage
GABA: gamma aminobutyric acid
LCM: lacosamide
LEV: levetiracetam
PER: perampanel
VPA: valproic acid
WHO: World Health Organization
